# Nucleoside Triphosphates — Building Blocks for the Modification of Nucleic Acids

**DOI:** 10.3390/molecules171113569

**Published:** 2012-11-15

**Authors:** Marcel Hollenstein

**Affiliations:** Department of Chemistry and Biochemistry, University of Bern, Freiestrasse 3, CH-3012 Bern, Switzerland; Email: hollenstein@dcb.unibe.ch; Tel.: +41-316-314-372

**Keywords:** modified nucleoside triphosphates, DNA polymerases, SELEX, DNAzymes, PEX, chemically modified nucleic acids

## Abstract

Nucleoside triphosphates are moldable entities that can easily be functionalized at various locations. The enzymatic polymerization of these modified triphosphate analogues represents a versatile platform for the facile and mild generation of (highly) functionalized nucleic acids. Numerous modified triphosphates have been utilized in a broad palette of applications spanning from DNA-tagging and -labeling to the generation of catalytic nucleic acids. This review will focus on the recent progress made in the synthesis of modified nucleoside triphosphates as well as on the understanding of the mechanisms underlying their polymerase acceptance. In addition, the usefulness of chemically altered dNTPs in SELEX and related methods of *in vitro* selection will be highlighted, with a particular emphasis on the generation of modified DNA enzymes (DNAzymes) and DNA-based aptamers.

## 1. Introduction

The importance of the role played by (2'-deoxy)ribonucleoside triphosphates [(d)NTPs] in numerous biological processes needs not to be further underscored. For instance, natural (d)NTPs serve as the fundamental building blocks in the polymerase-mediated synthesis of nucleic acids, both *in vitro* and *in vivo*, while ATP acts as the universal unit of molecular currency. Unsurprisingly, modified triphosphates have been the target of numerous synthetic campaigns since they represent the final and active form of DNA polymerase and reverse transcriptase inhibitors [1]. Moreover, since the advent of the polymerase chain reaction (PCR), numerous engineered (thermostable) DNA polymerases have been crafted [2]. Unlike their wild-type counterparts, these DNA polymerase variants possess a much broader substrate tolerance allowing for the polymerization of modified dNTPs and thus, for the generation of modified oligonucleotides and functional nucleic acids for a wide-ranging palette of applications [3].

This review article will focus on the recent progress made in nucleoside triphosphate synthesis and will highlight a few of the most prominent applications, including the use of modified dNTPs both as probes for unraveling the mechanism of polymerases, and in systematic evolution of ligands by exponential enrichment (SELEX) and related combinatorial methods of *in vitro* selection for the development of functional nucleic acids (*i.e.*, ribozymes, DNAzymes, and aptamers) [4,5,6].

## 2. Synthesis of Modified dNTPs

Even though a generally applicable and high-yielding method for the generation of dNTPs remains elusive, and despite the tedious purification that these reactive species must undergo, recent advances have certainly facilitated access to modified nucleoside triphosphates [7].

### 2.1. Yoshikawa Protocol

**Scheme 1:**
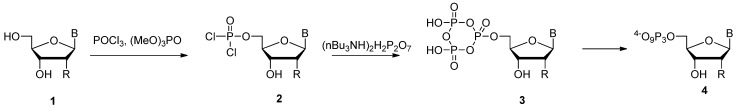
Yoshikawa method for the synthesis of nucleoside triphosphates (B = modified or natural nucleobase; R = H, OH, or modification).

One of the first, and still one of the most popular methods for the synthesis of nucleoside triphosphates is the Yoshikawa method (Scheme 1) [8,9]. This procedure involves the selective 5'-monophosphorylation of an unprotected nucleoside precursor **1** with the electrophilic phosphorous oxychloride (POCl_3_), yielding the highly reactive phosphorodichlorate intermediate **2**. This intermediate is then subsequently reacted *in situ* with pyrophosphate to yield the cyclic triphosphate **3**, which in turn is hydrolyzed to the desired compound **4**. 

The advantages of this methodology emanate from its simplicity. Indeed, no protecting group is required and the use of trialkylphosphate solvents mainly directs the phosphorylation to the 5'-regioisomer [7,10,11]. Consequently, a vast array of dNTPs modified with functionalities such as organic polymers [12], diamondoid-like structures [13], amino acid and amino acid-like residues [14,15,16,17,18,19,20,21], modified dNTPs with an sp^3^-hybridized carbon connecting the nucleobase and the linker arm [22,23,24], perfluorinated side-chains [25], unnatural bases [26,27,28,29], boronic acids [30], 2'-methylseleno triphosphates [31,32], and dual modified 4'-*C*-aminomethyl-2'-*O*-methylthymidine [33] have been reported. However, the use of a strong electrophilic phosphorous reagent is not compatible with all nucleosides [34], and modern analytical techniques have revealed the formation of a quantity of undesirable by-products [35].

### 2.2. Ludwig-Eckstein Method

**Scheme 2:**
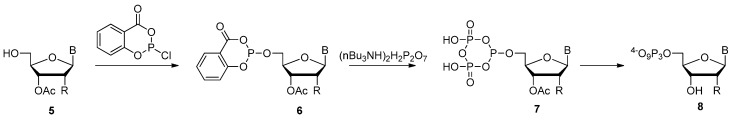
Ludwig-Eckstein synthetic approach (B = modified or natural nucleobase; R = H, OH, OAc or modification).

The “one-pot, three-steps” method developed by Ludwig and Eckstein in the late 80s, is still amongst the most reliable and popular procedures for the synthesis of modified triphosphates [36]. Briefly, the suitably 3'-*O*-protected (and 2'-*O* in the case of NTPs) modified nucleoside precursor **5** (see Scheme 2) is reacted with salicyl phosphorochlorite, which is active enough to specifically react with the free 5'-hydroxyl group to yield the activated phosphite intermediate **6**. The bifunctional phosphite **6** then undergoes two nucleophilic substitution reactions triggered by tris(tetra-*n*-butylammonium) hydrogen pyrophosphate, which leads to the displacement of salicylic acid and the formation of the cyclic intermediate **7**[36]. Finally, the iodine-mediated oxidation of derivative **7** yields the modified (d)NTP **8** via a cyclic nucleoside triphosphate.

**Figure 1:**
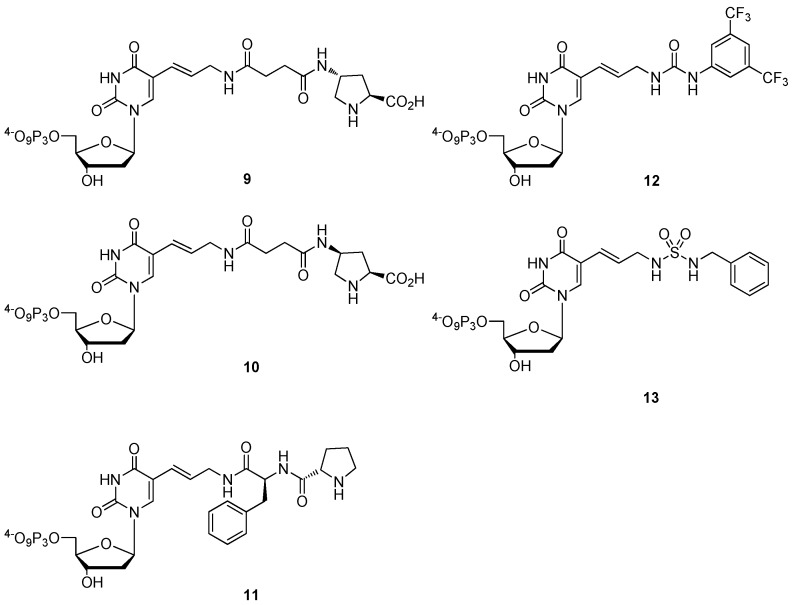
Chemical structures of the proline-containing analogues dU*^t^*^P^TP (**9**); dU*^c^*^P^TP (**10**); and dU^FP^TP (**11**); the urea modified dU^Bpu^TP (**12**); and the sulfonamide functionalized dU^Bs^TP (**13**) [51].

The Ludwig-Eckstein protocol has the advantage of reducing the amount of undesired by-products (e.g., regioisomers, mono-, di-, and oligo-phosphates) that are generated in the Yoshikawa methodology, which in turn drastically simplifies the ensuing HPLC purification [37]. In addition, the reaction can easily be followed by ^31^P-NMR and thus, despite being a one-flask protocol, the formation of all the intermediates can be monitored [36,38,39]. The only disadvantage of this protocol is the slightly longer synthetic route, especially when compared to the Yoshikawa method, since the free nucleoside needs first to be tritylated, then 3'-*O*-acetylated, before the DMTr protecting group can be unmasked to yield the precursor **5**. Despite this slight drawback, an impressive palette of modified dNTPs has been generated by application of the Ludwig-Eckstein protocol. Indeed, dNTPs adorned with amino acid-like side chains [38,40,41], α-L-threofuranosyl nucleoside triphosphates (tNTPs) [42,43], locked nucleic acid NTPs [44,45,46,47] and unnatural bases [48,49,50] have been generated. More recently, five modified deoxyuridine triphosphate derivatives (see Figure 1) bearing side-chains capable of organocatalysis (*i.e.*, bearing proline, urea, and sulfonamide groups) were synthesized and shown to be fully compatible with *in vitro* selection protocols since these dNTPs acted as substrates for polymerases in both primer extension reactions and in PCR [51].

### 2.3. The Borch Approach and Other Strategies

**Scheme 3:**
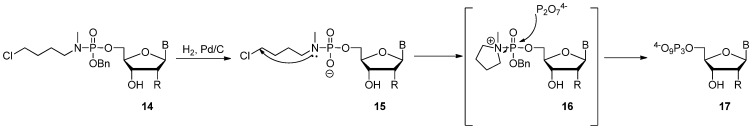
The Borch approach for the synthesis of nucleoside triphosphates (B = modified or natural nucleobase; R = H, OH, or modification).

In order to circumvent the drawbacks associated with other methods, *i.e.*, low yields, formation of side products, and incompatibility of functional groups with the strong electrophilic phosphorous reagents, an alternative strategy that employs a highly reactive zwitterionic intermediate has been developed (Scheme 3) [52]. Indeed, the *O*-benzyl-protected phosphoramidate ester **14** is activated by removal of the protecting group (usually by means of catalytic hydrogenation), which then leads to the formation of the reactive pyrrolidinium phosphoramidate zwitterion **16**. Intermediate **16** is prone to react *in situ* with a nucleophile in general, and pyrophosphate in particular. This approach was successfully applied in the synthesis of farnesyltransferase inhibitors [53], and phosphoramidate prodrugs [54]. However, this methodology has not been used for the development of modified dNTPs as yet, probably because of the rather sinuous synthetic route leading to intermediate **14**, an issue that has recently been addressed by the direct activation of more readily accessible *H*-phosphonate nucleosides [55].

**Scheme 4:**
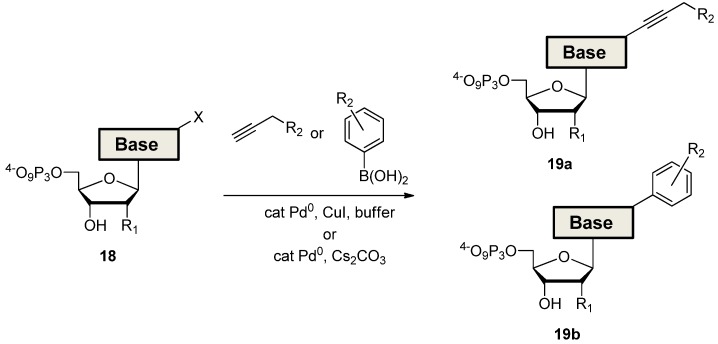
Synthesis of modified dNTPs via direct aqueous Sonogashira (compound **19a**) or Suzuki (compound **19b**) coupling reactions (R_1_ = H, OH, or modification; R_2_ = functional group) [56].

In a completely different approach, halogenated nucleoside triphosphates are used in direct aqueous cross-coupling reactions for the synthesis of modified dNTPs (Scheme 4) [56]. This convenient strategy, which completely bypasses the traditional multi-step procedures, has successfully been applied for the generation of dNTPs bearing a vast array of functional groups ranging from functional tags [57,58,59,60,61,62,63,64,65,66] and bile acids [67] to amino acids [68,69]. Finally, amide bond formation reactions were extensively used to connect dNTPs equipped with amine residues to side-chains bearing carboxylic acid groups, so as to yield triphosphates adorned with amino acid-like functionalities [14] or metal complexes [70,71].

## 3. Applications of Modified dNTPs

The enzymatic polymerization of chemically altered dNTPs represents a milder and alternative way for the synthesis of oligonucleotides, especially when compared to more traditional methods for the generation of modified nucleic acids such as solid-phase synthesis using phosphoramidite building blocks or post synthetic approaches [72,73]. Moreover, since modified dNTPs have been engaged in a motley array of applications such as the generation of modified oligonucleotides by the bias of the Nicking Enzyme Amplification Reaction (NEAR) [74,75], only a few will be considered in this section and the interested reader is directed to other excellent reviews dealing with other facets of modified dNTPs [3,56,72,76,77,78,79].

### 3.1. As Probes for Polymerases and Substrate Acceptance

Numerous modified dNTPs have been shown to be accepted as substrates and incorporated by DNA polymerases. In this context, the modifications are usually anchored at the C5 positions of pyrimidines and at the C7 of 7-deazapurines by means of rather rigid alkyne- or alkene-based linker arms. The polymerases seem to be quite tolerant to the nature of the chemical alteration appended, since both small substituents [25,80], as well as bulky groups [81], do not reduce the acceptance of the nucleosides. On the other hand, minute alterations in the structure of the linker arm, for instance substituting an *E*- for a *Z*-alkene, can have drastic and deleterious effects on the substance abilities of the dNTPs [19,82]. This clearly demonstrates that the prediction of the acceptance of dNTPs by polymerases is hazy and the underlying mechanisms are still not well understood, even though both factors are intrinsic for the rational design of modified triphosphates and the engineering of new polymerases with extended substrate tolerance [2,83]. In order to investigate the mechanisms that dictate DNA polymerase substrate selectivity and acceptance, suitably modified dNTPs were employed [84].

**Figure 2:**
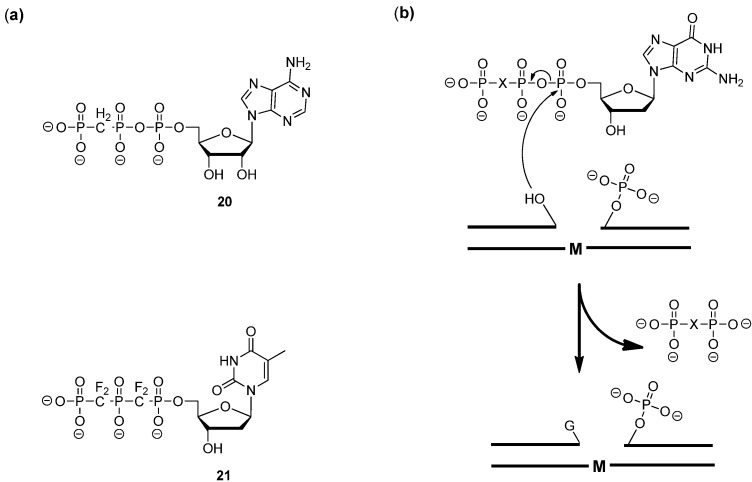
(**a**) Chemical structures of AMP-PCP **20** and (α,β)(β,γ)-bisCF_2_ dTTP **21**; (**b**) Schematic representation of the single-turnover kinetic assay using the modified dGTP and DNA polymerase β (X = CH_2_, CHF, CF_2_, CCl_2_, or O; M = C or T) [87].

In this context, non-hydrolysable dNTP substrate analogues in which one or all the bridging oxygen atoms of the triphosphate residue are replaced by methylene or other alkyl units, have been the target of numerous synthetic campaigns starting in the early 60s when 5'-adenylyl methylenediphosphonate (AMP-PCP) **20** (Figure 2a) was first synthesized [85]. Substitution of the β,γ-oxygen atom for a methylene linkage in **21** had only a limited impact on the leaving group efficiency of the corresponding pyrophosphate in RNA polymerase mediated reactions [86]. Moreover, a crystal structure of the DNA polymerase (pol) β with (β,γ)-CH_2_ dGTP revealed that the triphosphate region of both the modified analogue and the native dGTP could be superimposed with no significant deviation, suggesting that the active site was not perturbed by such a modification [87]. However, single-turnover kinetic assays (Figure 2b) revealed that despite the lack of structural disturbance, the nature of the substitution had a profound impact on the pol β-mediated nucleotidyl transfer efficiency and mechanism [87]. Indeed, replacement of the β,γ-bridging oxygen by CF_2_, CHF, CH_2_, and CCl_2_ units (X in Figure 2b) revealed a strong correlation between the rate constants for the slowest nucleotide insertion step (*k*_pol_) and the p*K*_a_ values of the corresponding bisphosphonates or pyrophosphate (when X = O), suggesting that the altered leaving groups had an impact on a chemical step rather than on the conformation of the enzyme. More in particular, the lower p*K*_a_s corresponding to the more electronegative bridges (X = CF_2_ and O), could stabilize the build-up of negative charge at the α,β-bridging oxygen during bond breaking and thus induce higher *k*_pol_ values. These findings further underscored that halogenated, especially fluorinated, bridging methylene residues were better surrogates than the original (β,γ)-CH_2_ analogues [88,89]. Recently, individual β,γ-CXY dNTP diastereomers could be synthesized and the absolute configuration at the chiral carbon was confirmed by X-ray crystallographic analysis of the complexes formed by the dNTP analogues and DNA pol β [90]. These analogues, especially the mono-fluorinated derivatives, can be useful probes for further investigating the mechanism of DNA polymerases [90,91]. Finally, (α,β)(β,γ)-bisCF_2_ substituted dNTP analogues were synthesized (**21** in Figure 2a) [92]. Unsurprisingly, these derivatives efficiently blocked DNA pol β in single-turnover gap-filling assays, proving their non-hydrolysable nature. More importantly, an X-ray structure of the complex formed by (α,β)(β,γ)-bisCF_2_ dTTP (**21**) and DNA pol β showed minimal distortion from the structure with the native dTTP [92].

DNA replication is an intricate and complex process, during which the polymerases switch from an “open” and catalytically inept form to a “closed” conformation that allows for the polymerization of the incoming triphosphate on the nascent chain [93,94]. Moreover, the incoming dNTP forms a Watson-Crick base pair with the templating nucleotide in the ternary complex formed by the DNA polymerase with the primer-template duplex. The Watson-Crick base pair is often complemented by hydrogen bonds and/or stacking interactions between the dNTP and some amino acid residues of the polymerase. It is thus of crucial importance to investigate the effect caused by base modification on the acceptance of dNTPs by DNA polymerases.

**Figure 3:**
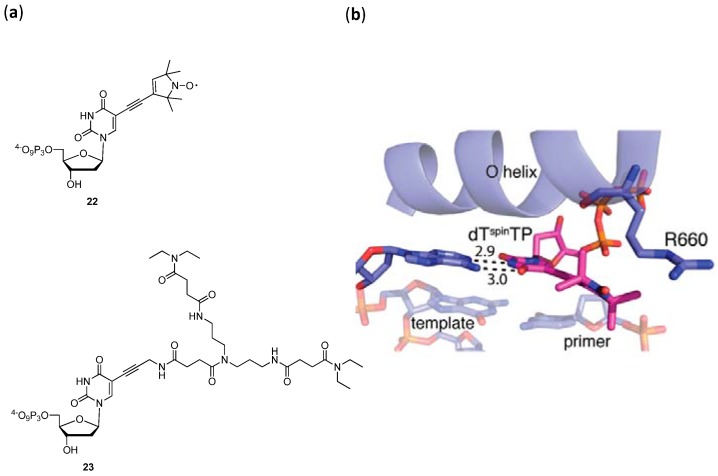
(**a**) Chemical structures of dT^spin^TP **22** and dT^dend^TP **23**; (**b**) Close-up view of the X-ray structure of the DNA polymerase *KlenTaq* with the modified triphosphate dT^spin^TP **22**, picture taken from reference [96].

In this context, two nucleoside triphosphates embellished with a nitroxide modification (dT^spin^TP **22** in Figure 3a) [95] and a flexible dendron (dT^dend^TP **23** in Figure 3a) [12] were first engaged in single nucleotide incorporation assays [96]. Both modified dNTPs are readily incorporated by the large fragment of *Thermus aquaticus* DNA polymerase (*KlenTaq*), albeit with a 2500- and 137-fold reduction in efficiency compared to the natural dTTP, respectively. The crystal structures of the modified dNTPs with *KlenTaq* bound to a primer-template duplex were obtained and both dT^spin^TP **22** and dT^dend^TP **23** caused only minor disturbances in the overall structure when compared to that of an unmodified dTTP. However, certain distinctions were apparent: in the structure of dT^spin^TP **22** (Figure 3b), Arg660 was in a different orientation in order to encompass the nitroxide residue, while in the structure with dT^dend^TP **23**, Arg660 interacts both with the phosphate-backbone and the amide moiety of the rigid propargylamide linker arm [96]. It was surmised that these subtle differences do account for the variation in the acceptance efficiency of both dNTPs.

Finally, in a recent report by Marx *et al.*, the polymerase acceptance of a series of aminopentinyl-modified nucleoside triphosphates was gauged at by incorporation assays and X-ray crystal structure analysis [97]. The polymerase *KlenTaq* presented a higher tolerance for the C7-modified 7-deazapurine analogues since they were incorporated with similar efficiencies compared to their natural unmodified counterparts, while the C5-derivatized pyrimidines caused a drop in the catalytic competence of the polymerase. The rather small flexible side-chains caused only minor disturbances in the X-ray structures of all the modified dNTPs trapped in the active site of *KlenTaq* when compared to that of a natural dTTP. Moreover, these modifications induced only a minor displacement of Arg660, especially when compared to the bulkier dNTPs **22** and **23**, which certainly explains their higher acceptance by the DNA polymerase [96,97].

### 3.2. Modified dNTPs and SELEX

While Nature is reluctant to leave DNA in a single-stranded form and rather compels it to the well-known double-helical structure, this constraint does not apply to chemists. This realization, in conjunction with the advent of the polymerase chain reaction, prompted Szostak *et al.*, Joyce *et al.*, and Gold *et al.* to develop a combinatorial methodology for the parallel screening of large populations of nucleic acid sequences, coined SELEX (systematic evolution of ligands by exponential enrichment) [4,5,98,99]. Application of this chemical variant of Darwinian evolution allowed for the generation of nucleic acids (aptamers) capable of selectively and tightly binding to specific targets [100] and of catalytic RNAs (ribozymes) and DNAs (DNAzymes) [101,102], all of which present an enormous potential for *in vivo* applications [103,104]. However, the use of natural nucleic acids imposes certain drastic restriction on the applicability of aptamers and nucleic acid enzymes gained through these *in vitro* selection experiments. Indeed, wild-type based DNA and RNA aptamers have quite a limited tolerance to nucleases and might endure chemical degradation [105]. Furthermore, the narrow chemical arsenal presented by nucleic acids, especially when compared to the wealth of functional groups endemic to proteins, restricts both the catalytic efficiencies and the range of reactions covered by DNAzymes (Dz) and ribozymes. In addition, natural nucleic acid enzymes often have to rely on external cofactors such as divalent metal cations (M^2+^) to achieve reasonable catalytic activities [106]. Consequently, the paucity of functional groups and the insignificant nuclease-resistance of wild-type nucleic acids, prompted the development of SELEX and related methods of *in vitro* selection using modified triphosphates [3,105]. Furthermore, the *in vitro* selection protocol involving modified dNTPs for the generation of DNA aptamers or DNAzymes is less cumbersome and time consuming than the RNA equivalent since the transcription and reverse transcription steps can be omitted. Thus, this part of the review will essentially deal with *in vitro* selections of DNA molecules.

#### 3.2.1. Selections of Modified DNAzymes

**Figure 4:**
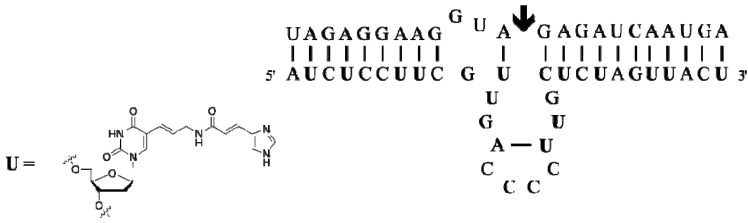
Sequence and hypothetical 2D structure of Dz16.2-11 (bold-face **U**’s indicate the position of the modified nucleoside; the arrow shows the cleavage site within the RNA substrate) [109].

Since the discovery of the first Pb^2+^-dependent RNA-cleaving DNAzyme [107], numerous deoxyribozymes have been identified, including the very potent Dz10-23 and 8-17 [108]. However, it was soon recognized that in order to alleviate the strong M^2+^-dependence of DNAzymes that is often not compatible with *in vivo* applications and in view of replenishing natural DNAs with functionalities capable of promoting catalysis, modified dNTPs had to be used in selection experiments. In this context, Dz16.2-11, a Zn^2+^-dependent RNA-cleaving DNAzyme, was isolated by *in vitro* selection using a C5-imidazole-functionalized dUTP that was used *in lieu* of its natural counterpart (Figure 4) [109]. In its minimal composition, Dz16.2-11 requires the presence of three essential imidazole modifications for optimal catalytic activity (*k*_cat_ > 1 min^−1^). These imidazole moieties are probably chelating the aminophilic Zn^2+^ and thus promoting bond cleavage via a mechanism reminiscent of protein enzymes such as carboxypeptidase A [109]. The rather short Dz16.2-11 presents a high catalytic efficiency (*k*_cat_/*K*_M_ ~ 10^8^ M^−1^min^−1^) under simulated physiological conditions (10 μM Zn^2+^, 1 mM Mg^2+^, 150 mM Na^+^, pH 7.5, at 37 °C).

**Figure 5:**
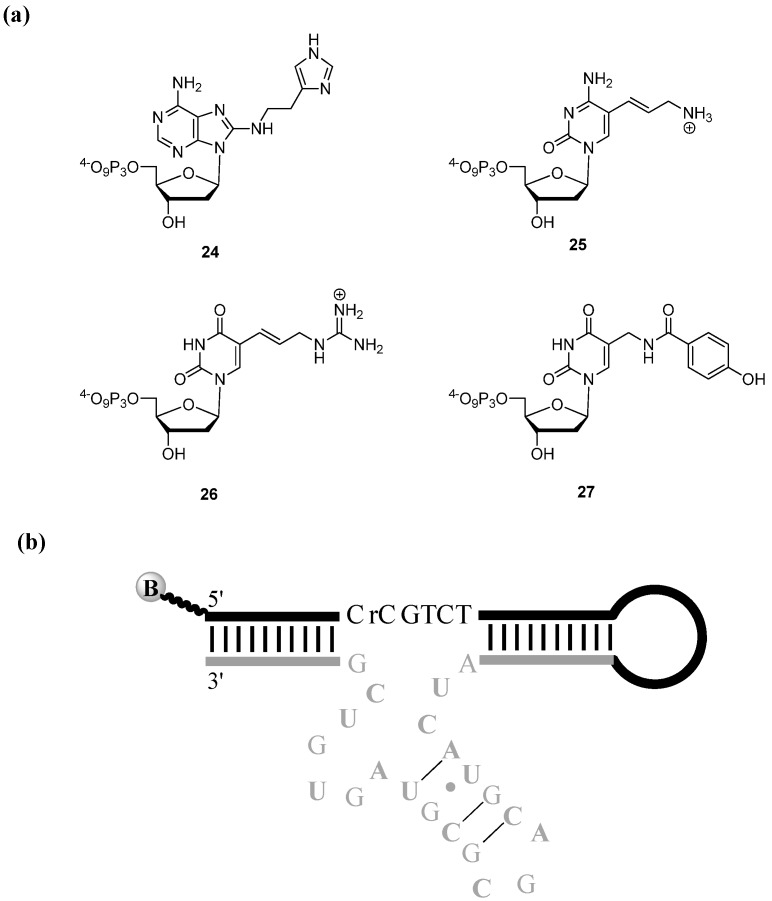
(**a**) Chemical structures of dA^im^TP **24**, dC^aa^TP **25**, dU^ga^TP **26**, and the phenol-modified dUTP **27**; (**b**) Sequence and hypothetical 2D structure of Dz9-86 (bold-face A’s, U’s, and C’s indicate the position of the modified nucleosides) [112].

Other *in vitro* selection efforts focused on the generation of M^2+^-independent ribophosphodiester-cleaving DNAzymes using a combination of dNTPs equipped with amino acid-like residues to compensate for the absence of M^2+^-cofactors [110,111,112,113,114]. Most notably, dA^im^TP **24** (Figure 5a) [115] along with the commercially available allylamino-dUTP (dU^aa^TP) were used conjunctly in an *in vitro* selection experiment that culminated with the identification of the RNA-cleaving DNAzyme 9_25_-11 [110]. Indeed, the cationic amine and the imidazole residues act in synergy through a general acid and base mechanism and convey robust catalytic activity to the self-cleaving Dz9_25_-11*c* (*c* for *cis*) in the absence of M^2+^ (*k*_obs_ ~ 0.2–0.3 min^−1^). Furthermore, Dz9_25_-11*c* could successfully be converted into a small 31 nucleotide *trans*-cleaving species, Dz9_25_-11*t*, which presented an appreciable catalytic efficiency (*k*_cat_/*K*_M_ ~ 510^5^ M^−1^min^−1^) under multiple turnover, again in the absence of M^2+^ [116,117,118]. In a similar attempt to mimic the active site of RNase A, Sidorov *et al.* simultaneously applied a C7-dATP analogue embellished with a cationic amine, in combination with a dUTP nucleotide bearing an imidazole function anchored at position C5 of the nucleobase in an *in vitro* selection [111]. The resulting DNAzyme employs the two protein-like residues for the M^2+^-independent and sequence-specific cleavage of a 12nt-long all-RNA substrate. While the first-order rate constants remain modest (*k*_obs_ ~ 0.07 min^−1^) when compared to protein enzymes, the selected DNAzyme is ~50-fold more proficient at promoting the cleavage of a ribophosphodiester linkage than unmodified DNAzymes in the absence of M^2+^ and other cofactors [111,119].

All the aforementioned RNA-cleaving modified DNAzymes induce impressive rate enhancements when compared to the uncatalyzed scission of ribophosphodiester linkages [120], but they are still inferior catalysts than their protein counterparts. Consequently, it was surmised that increasing the chemical space that can be explored during *in vitro* selections could possibly improve the catalytic efficiency of DNAzymes. By the same token, the selected DNAzymes were deemed to be sequentially different from known nucleic acid enzymes, thus avoiding the so-called “tyranny of the small motif” effect, which is known to have poisoned numerous selections [6,121]. Consequently, in order to expand the chemical landscape available for exploration during a selection experiment, a third modified dNTP was included in the process. Indeed, dU^ga^TP **26** (Figure 5a) was equipped with a guanidinium functionality that mimics the amino acid arginine and was thus expected to help stabilizing secondary and tertiary structures through its cationic nature [112]. This modified deoxyuridine derivative was used in a selection experiment for the generation of M^2+^-independent RNA-cleaving DNAzymes along with the analogues dA^im^TP **24** and dC^aa^TP **25** (Figure 5a) bearing imidazole and cationic amine residues, respectively. The resulting highly functionalized DNAzyme, Dz9-86 (Figure 5b), catalyzes the cleavage of a single embedded ribo(cytosine)phosphodiester bond with a rate constant for self-cleavage that was comparable to that of Dz9_25_-11*c* (*k*_obs_ = 0.13 min^−1^) [112]. However, unlike Dz9_25_-11, the catalytic activity of Dz9-86 increased with the temperature before reaching an apparent maximum at 37 °C. This temperature dependence along with the indifference of the catalytic rates on variation of the ionic strength was attributed to the additional stabilisation of the secondary and tertiary structures conveyed by the extra guandinium residues. Finally, even though Dz9-86 was selected for the cleavage of a single embedded RNA linkage, cleavage of 12 nt long RNA and 2'OMe substrates was also achieved, albeit with a ~100-fold drop in catalytic efficiency. While Dz9-86 represents a significant improvement compared to other modified DNAzymes, especially in terms of the specific catalytic M^2+^-independent scission of RNA linkages, this catalytic nucleic acid still presented some shortcomings, including rather low rate constants especially with all-RNA substrates and absence of a *trans*-cleaver that is necessary for practical applications [113].

In an effort to cover both wider chemical and sequence space and at the same time avoiding the tyranny of the small motif, the very same modified dNTPs as used in the selection for Dz9-86 (*i.e.*, dA^im^TP **24**, dC^aa^TP **25**, and dU^ga^TP **26**) were applied in combination with a larger randomized domain (N40 rather than N20) in a selection experiment. The resulting Dz10-66*c* displayed improved kinetics for self-cleavage in the absence of M^2+^-cofactors (*k*_obs_ = 0.5 min^−1^) when compared to both Dz9-86 and Dz9_25_-11*c* and was more effective at higher temperatures [113]. Moreover, Dz10-66*c* displayed a robust catalytic activity in a minimal buffer (*k*_obs_ = 0.1 min^−1^ in 1 mM EDTA, 5 mM NaHPO_4_ pH 7.4), conditions that are usually incompatible with nucleic acid mediated catalysis. These favorable assets were partially attributed to the stabilizing effect induced by the guanidinium groups. Finally, Dz10-66*c* could be converted to a *trans*-cleaving species Dz10-66*t* by means of a slight modification of the primer extension reaction protocol. Dz10-66*t* showed catalytic efficiencies under multiple turnover conditions (*k*_cat_/*K*_M_ ~ 610^5^ M^−1^min^−1^) that compared favorably with certain unmodified DNAzymes, but were still much inferior to that of Dz16.2-11 (Figure 4) or Dz10-23 when assayed under their optimum working conditions (*i.e.*, 10 μM Zn^2+^ and 2–100 mM Mg^2+^, respectively).

As mentioned in Section 3.1, numerous factors govern the efficiency of polymerizability of modified dNTPs, including the nature and the positioning of the functional groups and/or the connecting side chains [19]. These factors seem also to strongly affect the extent of the catalytic enhancement that emanate from DNAzymes that were generated through *in vitro* selections with modified dNTPs. Indeed, the polymerase acceptance of dA^im^TP **24** remains virtually unaltered when the linker arm connecting the imidazole moiety to the nucleobase was shortened in size by one methylene unit, while a similar increase in size had a deleterious effect on the polymerizability [38]. In order to assess the effect of the size of the linker arm on the catalytic proficiency of DNAzymes, an *in vitro* selection was carried out [114]. More specifically, this selection experiment encompassed the simultaneous use of the modified analogues dC^aa^TP **25** and dU^ga^TP **26** in conjunction with dA^imm^TP, a close mimic of dA^im^TP **24** where the linker arm is shrunk by one CH_2_-unit, and eventually led to the isolation of Dz20-49 [114]. A marked depletion in terms of catalytic efficiency for the M^2+^-independent cleavage of a single embedded RNA linkage was observed for Dz20-49 (*k*_obs_ = 310^−3^ min^−1^) when compared to Dz10-66*c* which utilizes the slightly longer ethylamino-linker arm to support the imidazole residues. It is quite baffling that such a minute change in the chemical structure and composition could have such a drastic impact on the catalytic efficiency of a DNAzyme, and more generally on the outcome of an *in vitro* selection experiment. 

The inclusion of the dUTP analogue **27** equipped with a side-chain mimicking the amino-acid tyrosine (Figure 5a) in an *in vitro* selection protocol using a similar construct as had been used in the case of Dz9_25_-11, allowed for the isolation of Dz11-17PheO [122]. This DNAzyme self-cleaved an embedded ribo(cytosine)phosphodiester linkage with an appreciable rate-constant (*k*_obs_ > 0.2 min^−1^) when supported by the presence of either Ca^2+^, Zn^2+^, Mg^2+^ or Mn^2+^. 

Finally, an RNA amide synthetase [123] and a Diels-Alder ribozyme [124] were isolated by *in vitro* selection making use of a 5-imidazole modified UTP analogue and a UTP equipped with a pyridiylmethyl unit, respectively. The Diels-Alder ribozyme presented a strong requirement for the presence of Cu^2+^, while this transition metal only enhanced the catalytic efficiency of the RNA amide synthetase by changing the affinity of the RNA for its substrate. Both ribozymes induce significant rate enhancements when compared to the uncatalyzed reactions, and certainly help to broaden the catalytic repertoire of nucleic acid based enzymes. 

#### 3.2.2. Aptamer Selections

Since the advent of SELEX [4,5,98], a flourishing number of aptamers have appeared, propelling these nucleic acids into a leading class of molecular biosensors for a wide ranging diversity of analytes [125,126]. Besides their role as biosensors, aptamers serve in many other applications such as drug development, therapy, target validation, and functional characterization [100,127]. Despite this large success, aptamers consisting of wild-type DNA or RNA are subjected to nuclease degradation which is highly detrimental for numerous practical applications. Furthermore, the rather functionality deprived nucleic acids offer, besides intricate binding motifs, few chemical handles for the interaction with specific residues on the intended targets. Therefore, the chemical modification of aptamers for improving their nuclease-resistance and/or their binding affinities is an important and necessary improvement. Initially, aptamers were modified post-SELEX by capping the 3'- or 5'-termini or by introducing point-mutations (e.g., 2'-fluoronucleotides or LNAs) at various locations on a trial-and-error basis [127]. Nevertheless, as seen in Section 3.2.1 for DNAzymes, the inclusion and alteration of chemical functionalities in the sequence of an aptamer can result in a dramatic loss of binding affinity. Consequently, modified dNTPs and NTPs have advanced as convenient vectors for the elaboration of chemically altered aptamers. In this context, 2'-modified nucleoside triphosphates and thiophosphate analogues have found extensive usage due to their acceptance by RNA and DNA polymerases [79]. Due to the wealth of aptamers that have been crafted using the aforementioned modifications, this section will focus on only a few recent examples involving different NTPs and dNTPs and the interested reader is directed to other excellent reviews covering 2'-modified and thiophosphate nucleotide-based aptamers [79,100,105,127,128]. 

The first example of an aptamer *in vitro* selection using a modified dNTP was reported in 1999 [129]. Indeed, Benner *et al.* used a dUTP analogue carrying a cationic amine (connected to the nucleobase via a propynyl-linker arm) to select for ATP, ADP, and AMP binding aptamers. The resulting sequences bore few similarities to the motifs that were observed in the selection with wild-type triphosphates [130,131]. Unexpectedly, both the modified and the natural aptamers appeared to form bimolecular complexes with ATP and this with rather similar affinity constants (~110^−5^ M^2^), which was attributed to the influence of an external stimulus on the selection stringency, namely the effective concentration of the adenosine derivative bound to the solid support (~3 mM). Moreover, the sequences emanating from the selection with the modified dNTP were much shorter than those stemming from the selection with the natural triphosphates (25 *vs.* 69 nucleotides, respectively), a shrinking effect that has been observed in the selection of modified DNAzymes, albeit to a lesser extent [112,132]. Finally, substituting the amino-modified dU residues for their natural counterparts results only in a two-fold loss of binding affinity, suggesting that the chemical alterations only had a modest impact on the overall properties of the aptamers [129].

**Figure 6:**
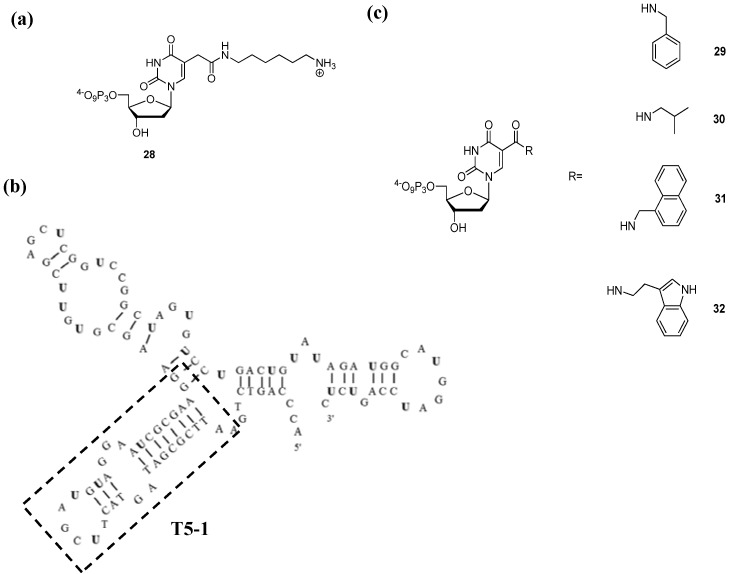
(**a**) Chemical structure of the amine-modified dUTP; (**b**) Sequence and hypothetical 2D structure of aptamer **T5** (bold-face U’s indicate the position of the modified nucleosides) [134]; (**c**) Chemical structure of the carboxamide-modified dUTPs [40].

An elegant landmark example was reported by Sawai *et al.* where a deoxyuridine triphosphate analogue adorned with a cationic amine connected via a hexamethylene linker arm to the C5 of the nucleobase (Figure 6a) [133] was used in an *in vitro* selection for thalidomide-binding aptamers [134]. After 15 rounds of selection, 44 individual clones were isolated. The most proficient binder, DNA aptamer **T5** (Figure 6b), displayed a dissociation constant (*K*_d_) of 113 μM for thalidomide. After dividing **T5** into three distinct domains, it could be shown that the **T5-1** region (square in Figure 6b) was mainly responsible for binding to the target compound since the *K*_d_ of a truncated version of **T5** with the exact sequence composition of **T5-1** was similar to that of the entire aptamer. Surprisingly, even though **T5** was selected with a racemic mixture of thalidomide, this aptamer showed high binding to the (*R*)-form of thalidomide and displayed no affinity to the (*S*)-enantiomer [134]. Finally, it was shown that the aptamer **T5** crucially depended on the presence of the modifications since the corresponding natural DNA sequence lost all binding-propensity. 

Various carboxamide-modified dUTP analogues were recently synthesized (Figure 6c) and shown to be good substrates for D. Vent and KOD XL DNA polymerases in primer extension reactions but were quite reluctant to amplification under PCR conditions [40]. In order to gauge at the effect of a side-chain on the outcome of an *in vitro* selection, these modified dNTPs were engaged in selections for aptamers binding to either the challenging human tumor necrosis factor receptor super family member 9 (TNFRSF9) or the tumor-associated calcium signal transducer 2 (TACSTD2) as a positive control [40]. These specific protein targets were chosen because no DNA aptamer (natural or modified) for TNFRSF9 has ever been selected despite various attempts, and TACSTD2 has a rather strong affinity (*K*_d_ < 100 nM) to random DNA pools [40]. After 8 rounds of selection, strong binding to TNFRSF9 was observed in the selections with dUTP **29** and **32**, while that with triphosphate **30** led to only poorly active aptamers (*i.e.*, with *K*_d_ > 100 nM). In the case of the control experiment, all the selections (TTP, **29**, **30**, and **32**) yielded aptamers with strong binding affinities to TACSTD2 (*K*_d_ ≤ 9 nM). Individual molecules were cloned from the population of the 8th round of the selection for TNFRSF9-binders using dUTP **32** and one single clone was further characterized. This specific aptamer, DNA clone 1684-40, showed a similarly high binding affinity for the target protein (*K*_d_~5 nM) than that of the entire population of the 8th generation. Finally, an enzymatic synthesis of DNA clone 1684-40 performed using TTP, dUTP **29**, and triphosphate **31** resulted in a total ablation of the binding affinity of the aptamers to TNFRSF9 and thus further highlighting the need for the modification and revealing that minute alterations in the chemical structures can have drastic consequences.

It is noteworthy mentioning that not all the selections with modified dNTPs have such positive outcomes as the examples outlined above. Indeed, the *in vitro* selection of aptamers against human thrombin using a triphosphate analogue adorned with a pentynyl-side chain [135] resulted in modified DNA molecules that had only moderate binding affinities (*K*_d_~0.4–1.0 mM) when compared to aptamers resulting from a selection with natural dNTPs (*K*_d_~25–200 nM) [136].

## 4. Conclusions

The synthesis of modified analogues of nucleoside triphosphates still remains a rather knotty task, mainly because of the rather intricate and extensive purification step that is required. Nonetheless, recent progress certainly greatly facilitates access to these interesting and promising derivatives. Analogues where the bridging oxygen atoms of the triphosphate unit have been replaced by substituted methylene linkages have vastly improved the knowledge on the kinetics and the mechanisms underlying the uptake of dNTPs by polymerases. Furthermore, the X-ray crystal structure of base-modified dNTPs trapped within the active site of DNA polymerases provided insight on the criteria governing polymerase uptake of triphosphates. Surprisingly, most of these modified dNTPs caused only minor disturbances in the overall crystal structures when compared to that of unmodified triphosphates, suggesting that subtle interactions are important for a good substrate acceptance.

dNTPs equipped with functionalities anchored on the nucleobases have been used in selection experiments for the generation of DNAzymes with enhanced catalytic properties. In this context, triphosphates embellished with amino-acid-like side chains have proven to be particularly proficient for the development of M^2+^-independent RNA-cleaving DNAzymes. These modified DNAzymes represent a significant improvement over nucleic acid enzymes obtained with natural DNA since they do not depend on M^2+^ or any other type of cofactors and can thus exert their catalytic activity in media that are not suitable for unmodified DNAzymes. Hopefully, the use of modified dNTPs in selection experiments will result in the generation of DNAzymes that present higher nuclease resistance, better catalytic activities (especially in media with low M^2+^ concentrations), and/or cellular delivery. In this context, LNA-modified DNAzymes have already shown promising properties [137,138,139]. Modified dNTPs have also extensively been employed in selection experiments for the generation of aptamers with improved binding affinities and/or nuclease resistance. In a landmark experiment, a DNA aptamer bound *enantioselectively* to the (*R*)-form of thalidomide, highlighting the usefulness of supplementing DNA with additional functionalities.

Finally, modified dNTPs are advancing as important building blocks for the generation of functionalized nucleic acids, especially in the context of *in vitro* selection experiments. However, numerous parameters including the size, the nature and the location of the modification on the nucleoside can dramatically change the outcome of a selection experiment and determine the level of polymerase acceptance of the modified dNTPs. Certainly, more chemical examples are needed to better understand all these underlying factors so as to improve the polymerase uptake and the efficiency of functionalized nucleic acids.
